# Fostering Teachers’ Work Engagement: The Role of Emotional Self-Efficacy Toward One’s Own Emotions, Professional Self-Efficacy, and Job Resources

**DOI:** 10.3390/ejihpe15090176

**Published:** 2025-08-29

**Authors:** Alessio Tesi, Andrea Baroncelli, Carolina Facci, Antonio Aiello, Enrica Ciucci

**Affiliations:** 1 Department of Political Science, University of Pisa, 56126 Pisa, Italy; alessio.tesi@unipi.it (A.T.); antonio.aiello@unipi.it (A.A.); 2 Department of Philosophy, Social Sciences and Education, University of Perugia, 06123 Perugia, Italy; 3 Department of Education, Languages, Intercultures, Literatures, and Psychology, University of Florence, 50135 Florence, Italy; carolina.facci@unifi.it (C.F.); enrica.ciucci@unifi.it (E.C.)

**Keywords:** emotional self-efficacy, professional self-efficacy, job resources, work engagement, teachers

## Abstract

The present cross-sectional study explored the synergic role of self-report measures of emotional self-efficacy toward one’s own emotions (i.e., the extent to which individuals perceive themselves as confident and effective in managing their emotions), professional self-efficacy, and job resources on work engagement among 589 in-service teachers coming from public kindergartens, elementary schools, and middle schools. A hierarchical linear regression approach including a three-way interaction analysis revealed that (a) the two forms of teachers’ self-efficacy were uniquely and positively associated with work engagement, and (b) emotional self-efficacy toward one’s own emotions was positively associated with work engagement, especially at low (vs. high) levels of both professional self-efficacy and job resources. The results were discussed framing the job demands–resources model and stressing the importance of taking care of both personal and professional teachers’ self-efficacy, especially when the school environment lacks job resources.

## 1. Introduction

This research was carried out to explorethe synergic role of emotional self-efficacy toward one’s own emotions and professional self-efficacy (i.e., two different forms of teachers’ self-efficacy) and job resources on work engagement (WE) among a large sample of in-service teachers. WE, defined as a “positive, fulfilling, work-related state of mind that is characterized by vigor, dedication, and absorption” (Schaufeli et al., 2002, p. 74), is one of the most recognized indicators of work-related well-being (Bakker & Demerouti, 2017). Studies conducted under the framework of the job demands–resources model (Bakker & Demerouti, 2017) indicated that WE is predicted by both personal resources (e.g., self-efficacy, self-esteem, optimism, and emotional intelligence) and work-related contextual resources pertaining to organizational dynamics (e.g., social support, quality of communication, and leadership; Bakker & Demerouti, 2017; Mussagulova, 2021; Tesi, 2021; Tesi et al., 2024). As for personal resources, starting from the notion that helping professions are highly exposed to emotional demands at work (Tesi, 2021), this research paid particular attention to considering two distinct forms of teachers’ personal resources pertaining their self-assessment of (a) professional competence and (b) personal emotional competence (i.e., emotional self-efficacy toward their own emotions).

### 1.1. Teachers’ Self-Efficacy and Work Engagement

A growing body of recent research on teachers’ individual characteristics has recognized a close association between teachers’ professional functioning (e.g., teaching quality and relationship management skills at school) and their personal socio-emotional functioning (e.g., physical health and subjective well-being, and personal socio-emotional competence; Jennings & Greenberg, 2009; Schelhorn et al., 2023). Specifically, when teachers face their multiple daily tasks (e.g., they carry out teaching activities, relate to students, colleagues, and students’ families, dealing with bureaucracy and legal responsibilities, etc.), on the one hand, they implement technical–professional skills, which are the result of the experience gained during their education and working career; on the other hand, they are activated on an emotional dimension that involves individual characteristics pertaining to their personal lives that go beyond the boundaries of their professional role (Jennings & Greenberg, 2009). This is in line with the literature indicating that all people working in helping professions are particularly exposed to emotional demands at work and require highly developed emotional competencies (Tesi, 2021).

With the aim of exploring how different teachers’ individual characteristics are associated with WE, in this study we framed Bandura’s construct of self-efficacy (i.e., the subjective perceptions and beliefs of one’s own capability to perform specific activities or gain desired goals; Bandura, 1997; Lippke, 2020). Due to its domain-specific nature, we focused on two distinct forms of teachers’ self-efficacy: professional self-efficacy in relation to their role as teachers (i.e., a work-related personal resource) and emotional self-efficacy in relation to the management of their own emotions (i.e., a personal resource regarding the ability to manage one’s own emotions across life domains). Specifically, professional self-efficacy is defined as the set of perceptions and beliefs concerning one’s own ability to successfully cope with tasks and challenges related to one’s own professional role (Caprara et al., 2006); existing research has found bidirectional positive associations between teachers’ professional self-efficacy and WE (Burić et al., 2022; Laguna et al., 2017; Zee & Koomen, 2016): at first glance, self-efficacy drives people’s efforts in tasks to attain goals; thus, higher levels of professional self-efficacy are associated with greater levels of vigor, dedication, and absorption toward work. On the other hand, since higher levels of WE are associated with higher levels of job performance and pleasant emotions, this condition serves as a source of information to develop or strengthen positive self-efficacy beliefs. With reference to emotional self-efficacy, this construct defines the degree to which people self-perceive as confident and efficacious with their emotional abilities (Dacre Pool & Qualter, 2013; Kirk et al., 2008); even though there is scarce research focused on emotional self-efficacy and work contexts, a positive relationship with WE is expected based on previous studies highlighting that higher levels of emotional self-efficacy are associated with higher positive mood and lower negative mood, better management of the negative effects of anxiety, higher academic success, and graduate employability (Dacre Pool & Qualter, 2013; Kirk et al., 2008; Qualter et al., 2012). Moreover, existing results suggest that emotional competence (i.e., a broader construct strictly related to emotional self-efficacy) is associated with higher levels of WE and lower levels of burnout (Tesi, 2021). It is worth noting that we considered a measure of emotional self-efficacy explicitly focused on how people deal with their own emotions in everyday life (i.e., the whole construct of emotional self-efficacy also includes how people self-perceive as efficacious in dealing with others’ emotions); this allowed us to reduce the overlap with professional self-efficacy, that—as mentioned before—concerns the beliefs pertaining to professional efficacy in teaching’s daily tasks.

**Figure 1:**
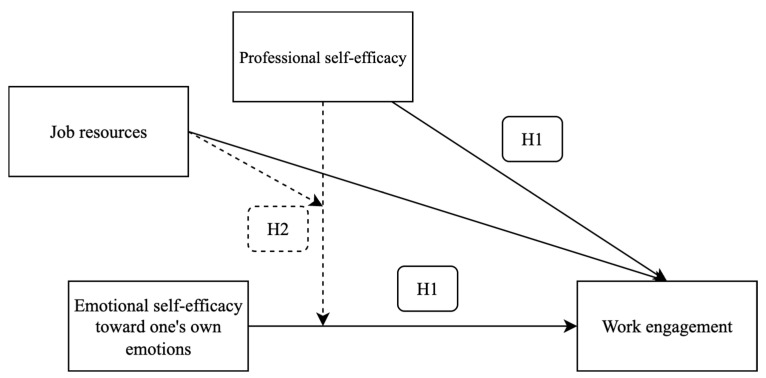
Graphical model of hypotheses. Note: continuous lines refer to the direct paths of H1; discontinuous lines refer to the interaction hypothesis (H2).

To sum up, as far as we know, the association between the two forms of self-efficacy and WE presented here has not been simultaneously tested. Considering the notion that both professional and emotional self-efficacy are two relevant distinct personal resources for the teaching profession (Jennings & Greenberg, 2009; Schelhorn et al., 2023), we tested the following hypothesis:

**H1:**  *We hypothesized that professional self-efficacy and emotional self-efficacy uniquely contribute to teachers’ WE (Figure 1).*

It is important to note that we tested the contribution of these two variables to WE while controlling for years of professional experience, which has been shown to influence teacher well-being, particularly in interactions with emotional competencies (Fernández-Berrocal et al., 2017).

### 1.2. The Role of Job Resources

Within the job demands–resources model (Bakker & Demerouti, 2017), professional self-efficacy and emotional self-efficacy toward one’s own emotions can be classified as personal resources (i.e., workers’ individual characteristics that help in gaining control of environments and achieving desired goals). Personal resources play a crucial role in enhancing employees’ WE, particularly in demanding work environments or in contexts lacking sufficient job resources (Bakker et al., 2007; Bakker & Sanz-Vergel, 2013; Tesi et al., 2024).

To account for this, in the present study we also considered teachers’ perception of the job resources in their workplace, that is, the organizational dynamics that are functional to reaching work goals, promoting workers’ personal growth, and reducing the impact of work stressors. Job resources include all those opportunities that drive human resource development (i.e., the organizational climate with colleagues and superiors, career opportunities, etc.; Bakker & Demerouti, 2017). In detail, job resources constitute both intrinsic and extrinsic motivational factors (Bakker et al., 2007) that make teachers more likely to develop and strengthen WE (Bakker & Demerouti, 2017). Our prediction was that the perception of low job resources (i.e., job resource deprivation) makes the contribution of teachers’ personal resources more salient. In particular, in the context of this study, we conceptualized the self-evaluation of one’s ability to manage their own emotions (i.e., emotional self-efficacy toward one’s own emotions) as a broad-spectrum personal resource that exerts its most beneficial effect on WE when teachers are unable to draw on either their work-related personal resources (i.e., low professional self-efficacy) and their contextual job resources (i.e., low job resources). As per H1, we tested the association between the interaction among emotional self-efficacy toward one’s own emotions, professional self-efficacy, and job resources on WE controlling for years of professional experience (Fernández-Berrocal et al., 2017). To sum up, we advanced the following hypothesis:

**H2:**  *Emotional self-efficacy toward one’s own emotions is positively associated with WE especially at low (vs. high) levels of both professional self-efficacy and job resources (Figure 1).*

## 2. Materials and Methods

### 2.1. Participants and Procedures

Participants were a convenience sample of in-service teachers recruited on a voluntary basis. They came from 27 public comprehensive administrative institutions located in central Italy. A comprehensive administrative institution brings together public kindergartens, elementary schools, and middle schools of the same geographical area sharing formal regulations and educational practices. The research was presented to all the teachers of each institution through school meetings or official school channels; then each teacher could voluntarily decide whether to take part in the study, with no economic benefit. Informed consent was obtained from all participants, and data collection was realized through an online self-report tool. All procedures were approved by local scholastic institutions, and the study was conducted in accordance with the ethical principles of the Declaration of Helsinki for conducting research.

The final sample was made up of 589 teachers (mean teaching experience = 17.11 years, SD = 10.95 years): 198 (33.62%) worked in kindergartens, 247 (41.93%) in elementary schools, and 144 (24.45%) in middle schools; 265 (44.99%) participants held a high school diploma and 324 (55.01%) a university degree. As is common in the Italian school system, the vast majority of the participants were females (n = 555, 94.23%).

### 2.2. Measures

#### 2.2.1. Teachers’ Professional Self-Efficacy

Teachers’ professional self-efficacy was assessed using the 6-item self-report questionnaire provided by Caprara (2001). Teachers were asked to indicate their agreement with each item using a 5-point Likert-type scale, from “not at all” (1) to “very much” (5). Items were focused on tasks and challenges that teachers face at school (e.g., “I am able to integrate effectively with colleagues”; “I am able to successfully solve the problems posed by my work, even the most demanding ones”). A mean score of all items was calculated for each participant; Cronbach’s alpha in the sample was equal to 0.82.

#### 2.2.2. Teachers’ Emotional Self-Efficacy Concerning Their Own Emotions

Teachers’ emotional self-efficacy concerning their own emotions was investigated with a dimension of the Crèche Educator Emotional Style Questionnaire (CEESQ, Ciucci et al., 2015) adapted for use with teachers from kindergarten onwards. It is a 5-point Likert-type scale (from 1 = “not true” to 5 = “completely true”) made up of 10 items focused on processes concerning recognition, expression, and the regulation of one’s own emotions (e.g., “I can easily identify the reasons for my emotions”; “I am able to express what I feel”; “I am able to manage emotions that are too intense”). Cronbach’s alpha in the sample was 0.86.

#### 2.2.3. Job Resources

Teachers’ perception of job resources in their workplace was assessed using the job resources subscale of the Integrated Tool of Organizational Well-being Evaluation (Tesi & Aiello, 2021). This is a 7-point Likert-type scale (from 0 = “completely disagree” to 6 = “completely agree”) composed of 8 items adapted to be administered in a school context to assess the extent to which the organizational environment is perceived as resourceful (e.g., “In this school organization we maintain good communication”; “I trust how people are judged here”). Cronbach’s alpha in the sample was 0.86.

#### 2.2.4. Work Engagement

WE was assessed with the 17-item form of the Utrecht Work Engagement Scale (UWES-17; Schaufeli et al., 2002; Italian version by Pisanti et al., 2008). Using a 7-point Likert-type scale, from 0 (“never”) to 6 (“always/every day”), teachers expressed their agreement with items that refers to vigor, dedication, and absorption toward their work experience (e.g., “At my job, I feel strong and vigorous”; “I am enthusiastic about my job”; “I am immersed in my work”). Cronbach’s alpha in the sample was 0.93.

### 2.3. Analyses

After the inspection of descriptive statistics and Pearson’s zero-order correlations, study hypotheses were tested with a linear hierarchical regression approach. Specifically, to test H1, years of professional experience (i.e., as a covariate), professional self-efficacy, and emotional self-efficacy toward one’s own emotions were inserted in Step 1; to test H2, the dimension of job resources was added in Step 2 among all the possible 2-way interaction terms between professional self-efficacy, emotional self-efficacy toward one’s own emotions, and job resources, and their 3-way interaction term was added in Step 3. Prior to computing interaction terms, scores were centered by subtracting the sample means. To interpret the 3-way interaction term, a test for the significance of differences between slopes was conducted according to Dawson and Richter (2006).

## 3. Results

Descriptive statistics and Pearson’s correlations are reported in Table 1.

Results of regression analyses are reported in Table 2. Specifically, and according to H1, both professional self-efficacy (β = 0.29, *p* < 0.001) and emotional self-efficacy toward one’s own emotions (β = 0.26, *p* < 0.001) uniquely and significantly contributed to WE (see Step 1). Moreover, the 3-way interaction term tested in Step 3 was significant (β = 0.11, *p* < 0.05).

**Figure 2:**
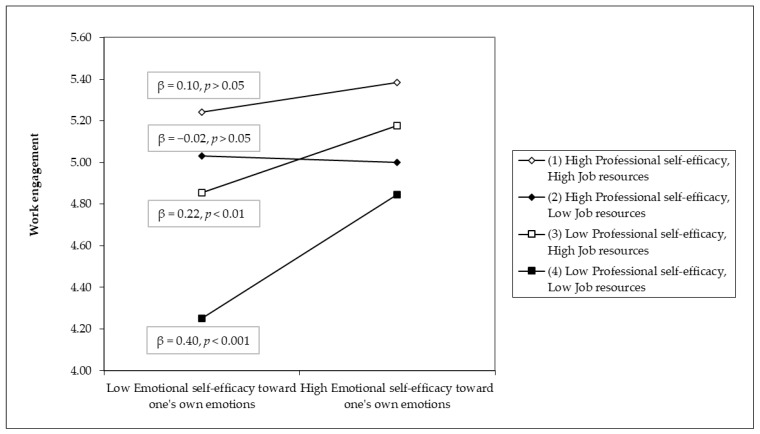
The 3-way interaction between emotional self-efficacy toward one’s own emotions, professional self-efficacy, and job resources on work engagement.

The test for differences between slopes (see Figure 2 and Table 3) indicated that, according to H2, the slope pertaining to the association between emotional self-efficacy and WE (β = 0.40, *p* < 0.001) at the conditional level of both low professional self-efficacy and low job resources was significantly different and stronger compared to the other three slopes. Moreover, the slope pertaining to low professional self-efficacy and high job resources was significantly different from the slope related to high professional self-efficacy and low job resources, thus indicating that emotional self-efficacy toward one’s own emotions contributed more to WE when levels of professional self-efficacy were low and job resources were high (β = 0.22, *p* < 0.01), rather than when professional self-efficacy were high and job resources were low (β = −0.02, *p* > 0.05).

## 4. Discussion

Within the framework of the job demands–resources model (Bakker & Demerouti, 2017; Mussagulova, 2021), the present study explored the synergistic role of two different forms of teachers’ personal resources, namely professional and emotional self-efficacy, and how they interact with contextual factors (i.e., job resources) to increase WE in a sample of in-service teachers. In fact, in line with the evidence that helping professions are highly exposed to emotional demands at work that can contribute to emotional fatigue (Tesi, 2021), existing literature on teaching highlights the importance of personal resources in helping teachers to cope with demanding and stressful school contexts (Jennings & Greenberg, 2009; Schelhorn et al., 2023). The results obtained in the present study confirmed our hypotheses and offered insights to inform both research and intervention practices. We found that both professional self-efficacy and emotional self-efficacy toward one’s own emotions were uniquely associated with WE. This result is in line with previous studies indicating that professional competencies and personal emotional competencies contribute to enhance WE (e.g., Burić et al., 2022; Laguna et al., 2017; Tesi, 2021; Zee & Koomen, 2016). Nevertheless, this evidence advances previous results since the significant role of each form of teachers’ self-efficacy on WE was unique (i.e., over and above the role of the other form). In other words, this result further highlights the stringent continuity between teachers’ personal life and professional role (Jennings & Greenberg, 2009), confirming that—over and above technical–professional preparation and years of experience—there are processes concerning individuals’ emotional functioning that account for their engagement toward work experience that should be central from both a theoretical and an educational point of view. Furthermore, the presence of the significant 3-way interaction term (i.e., indicating that the positive association between emotional self-efficacy toward one’s own emotions and WE is more salient—among all the possible combinations—when both professional self-efficacy and job resources are low) offers more in-depth considerations. For instance, this evidence is in line with previous results indicating that the contribution of personal resources, such as professionals’ self-efficacy, were positively related to WE when work contexts were particularly demanding (Bakker & Sanz-Vergel, 2013; Xanthopoulou et al., 2013), implying an increased use of personal resources to cope with contextual issues. In addition, this result can help to advance the understanding of how different facets of self-efficacy—acting as personal resources as per the theoretical framework of the job demands–resources model (Bakker & Demerouti, 2017)—help people to promote their WE: personal emotional competence seems to constitute a fundamental reservoir of resources for the teaching profession (Fernández-Berrocal et al., 2017), especially when teachers self-perceive low technical skills and can count on low resources within their work-context. In other words, teachers’ psychological processes concerning their personal emotional functioning should be necessarily taken into account both in teachers’ education and in the promotion of teachers’ well-being, as they might be essential factors in the resilience of these professionals. For example, a recent study by Gilar-Corbi et al. (2024) found that emotional intelligence was positively associated with psychological well-being in a sample of future teachers. However, the association between some of the components of emotional intelligence and psychological well-being became non-significant once resilience was introduced into a hierarchical model, indicating that resilience may function as a mediator: the capacity to manage emotions could foster resilience, which in turn promotes adaptive functioning among (future) teachers

The discussions reported above have to be read within the context of some limitations. First, the self-report nature of all instruments has allowed teachers’ subjective perception of the study variables to be reached, and it is important when variables related to self-efficacy or WE are assessed; a future study could consider other sources of information to assess job resources, since self-report perceptions of this construct could be biased reflecting teachers’ personal characteristics. Further, the cross-sectional research design limits the investigation of causality; a longitudinal research design might inform on causal mechanisms that in the present study can only be hypothesized. Moreover, the use of a convenience sample that comes from a single geographical area could impact the generalizability of the results that emerged. Finally, an in-depth exploration is needed to explain other evidence that emerged from the 3-way interaction term: emotional self-efficacy toward one’s own emotions was also associated with WE in the presence of low levels of professional self-efficacy and high job resources. It appears that emotional self-efficacy is a factor that promotes resilience primarily in the absence of another individual (rather than contextual) domain-affine variable. This may be due to the high impact of job resources on people’s work-related well-being, over and above personal resources (Bakker & Demerouti, 2017). However, future research should extend and clarify these findings, e.g., by also testing a measure of job demands.

## 5. Conclusions

Notwithstanding the limitations reported above, we believe that our findings may serve to advance both the theoretical and intervention approaches to improve the quality of teachers’ work experience. Although education and training practices for teachers did not significantly include attention to teachers’ personal characteristics until recent years (Jennings & Greenberg, 2009), our results indicate that promoting the skills needed for adaptive engagement in one’s work and its different tasks is a complex challenge. This challenge requires the combination of technical-professional skill acquisition and care for the teacher as a person. With specific attention to our study variables, we highlight that self-efficacies can be developed with appropriate training (Lippke, 2020). In this regard, our study stresses the appropriateness of proposing training courses for teachers that involve experiential activities that favor a representation of themselves as both professionals and individuals able to access their multiple potentials and personal resources and whose baggage of emotional experiences, beliefs, strengths, and difficulties is recognized and valued.

## Figures and Tables

**Table 1:** Descriptive statistics and correlations (Pearson’s r) among study variables.

	M	SD	Skew.	Kurt.	1	2	3	4	5
1—Years of professional experience	17.11	10.95	0.43	−0.76	−				
2—Work engagement	4.86	0.75	−1.32	2.43	−0.03	−			
3—Professional self-efficacy	3.64	0.55	0.17	−0.12	0.09 *	0.41 **	−		
4—Emotional self-efficacy toward one’s own emotions	3.78	0.52	−0.15	−0.08	0.06	0.40 **	0.48 **		
5—Job resources	3.82	1.14	−0.38	−0.23	−0.06	0.41 **	0.24 **	0.29 **	-

Notes. ** *p* < 0.001, * *p* < 0.05.

**Table 2:** Regression analyses (standardized β).

	Work Engagement
Step 1	*F*(3,588) = 56.278 **; R^2^ = 0.22
Years of professional experience	−0.07
Professional self-efficacy	0.29 **
Emotional self-efficacy toward one’s own emotions	0.26 **
Step 2	*F*(7,588) = 40.313 **; ΔR^2^ = 0.10, R^2^ = 0.32
Years of professional experience	−0.04
Professional self-efficacy	0.28 **
Emotional self-efficacy toward one’s own emotions	0.19 **
Job resources	0.29 **
Emotional self-efficacy toward one’s own emotions × Professional self-efficacy	−0.14 **
Professional self-efficacy x Job resources	−0.04
Emotional self-efficacy toward one’s own emotions × Job resources	−0.02
Step 3	*F*(8,588) = 36.359 **; ΔR^2^ = 0.01 *, R^2^ = 0.33
Years of professional experience	−0.05
Professional self-efficacy	0.26 **
Emotional self-efficacy toward one’s own emotions	0.17 **
Job resources	0.26 **
Emotional self-efficacy toward one’s own emotions × Professional self-efficacy	−0.15 **
Professional self-efficacy x Job resources	−0.06
Emotional self-efficacy toward one’s own emotions × Job resources	−0.02
Emotional self-efficacy toward one’s own emotions × Professional self-efficacy × Job resources	0.11 *

Notes. ** *p* < 0.001, * *p* < 0.05.

**Table 3:** Comparison between slopes concerning the 3-way interaction term (emotional self-efficacy toward one’s own emotions × professional self-efficacy × job resources) on work engagement (see Figure 2).

Pair of Slopes	Slope Difference	*t*-Value	*p*-Value
(1) and (2)	0.17	1.229	0.220
(1) and (3)	−0.17	−1.492	0.136
(1) and (4)	−0.44	−3.744	<0.001
(2) and (3)	−0.34	−2.201	0.028
(2) and (4)	−0.61	−4.414	<0.001
(3) and (4)	−0.27	−1.992	0.047

## Data Availability

There are no unpublished data available. The corresponding author can be contacted regarding this matter.
